# Effects of Dietary Resveratrol Supplementation on Growth Performance and Anti-Inflammatory Ability in Ducks (*Anas platyrhynchos*) through the Nrf2/HO-1 and TLR4/NF-κB Signaling Pathways

**DOI:** 10.3390/ani11123588

**Published:** 2021-12-18

**Authors:** Hao Yang, Yingjie Wang, Mengru Liu, Xiao Liu, Yihan Jiao, Sanjun Jin, Anshan Shan, Xingjun Feng

**Affiliations:** Laboratory of Molecular Nutrition, Institute of Animal Nutrition, Northeast Agricultural University, Changjiang Street 600#, Xiangfang District, Harbin 150030, China; yanghao951209@163.com (H.Y.); wangyingjie1993@hotmail.com (Y.W.); liumengruu@163.com (M.L.); liuxiao@neau.edu.cn (X.L.); yihanjiao11@163.com (Y.J.); Sanjunjin@163.com (S.J.); asshan@neau.edu.cn (A.S.)

**Keywords:** TLR4, Nrf2, hepatitis, inflammatory response, oxidative stress

## Abstract

**Simple Summary:**

This study clarified for the first time that the Nrf2 and NF-κB signaling pathways in ducks are related to the antioxidant and anti-inflammatory effects. For the first time, we found that dietary resveratrol (RES) effectively alleviated the inflammatory response caused by lipopolysaccharide (LPS) by reducing oxidative stress and endoplasmic reticulum stress, alternating the blood biochemical index, and restoring the destruction of hepatocyte morphology. The results of this study provide strong evidence that dietary RES improved the anti-inflammatory ability and the growth performance of ducks.

**Abstract:**

The aim of this study was to explore the effect of dietary resveratrol on the growth performance and anti-inflammatory mechanism in ducks. A total of 280 one-day-old specific pathogen-free male ducklings (*Anas platyrhynchos*) with an average body weight of 35 ± 1 g were randomly divided into two dietary treatment groups with different supplementation levels of resveratrol for growth performance experiments: R_0_ and R_400_ (0 and, 400 mg kg^−1^ resveratrol, respectively). At the age of 28 days, 16 ducks were selected from each treatment group and divided into four subgroups for a 2 × 2 factorial pathological experiment: R_0_; R_400_; R_0_ + LPS; R_400_ + LPS, (0 mg kg^−1^ resveratrol, 400 mg kg^−1^ resveratrol, 0 mg kg^−1^ resveratrol, 400 mg kg^−1^ resveratrol + 5 mg lipopolysaccharide/kg body weight). The results showed that resveratrol significantly improved final body weight and average daily gain (*p* < 0.01) and alleviated the lipopolysaccharide-induced inflammatory response with a reduction in IL-1β and IL-6 in the plasma and the liver (*p* < 0.05). Resveratrol improved mRNA levels of Nrf2 and HO-1 and decreased the mRNA levels of TLR4 and NF-κB in duck liver (*p* < 0.05). Dietary resveratrol can improve growth performance and reduce inflammation through the Nrf2/HO-1 and TLR4/NF-κB signaling pathways in duck.

## 1. Introduction

Many kinds of Gram-negative bacteria are widely distributed in feed, soil, and drinking water in the process of large-scale poultry farming and pose a serious threat to animal health [1,2]. As a primary pathogenic component of Gram-negative bacteria and a gut-derived endotoxin, LPS could translocate to the whole body, inducing liver inflammation and activating a systemic immune response [3]. Eventually, it leads to an inhibition of growth performance in animals [4]. The effects of LPS on the physiological condition of mammals have been extensively investigated. However, there have only been a few studies evaluating the effects of LPS on ducks as compared with broilers. Several studies have reported that LPS challenge could reduce growth performance and induce oxidative stress and immune response in ducks [5,6,7].

Various plant extracts have been reported that could take effect as immunomodulators and act as mediators in vivo due to their antioxidant and anti-inflammatory activity [8]. RES (3,4,5 trihydoxystilbene) is well known for its antioxidant ability and anti-inflammatory ability. Recently, many studies have suggested that a moderate concentration of dietary supplementation of RES as a feed additive can improve the growth performance and meat quality in poultry [9,10]. In addition, dietary supplementation of RES decreased the production of inflammatory cytokines, which is caused by heat stress, in duck jejunum [11]. For animals, the liver is a crucial organ for immunization. It plays an important role in maintaining the metabolism of the whole body and nutrient homeostasis [12]. Previous studies have reported that LPS-binding protein (LBP), which is secreted by the liver, was considered a key factor in various LPS-induced inflammatory reactions. The level of inflammatory factors in the liver tissue reflects the systemic health and immune system status [13].

The main feature of LPS-induced pathophysiology is the release of numerous pro-inflammatory cytokines and the infiltration of neutrophils [14]. As one of the Toll-like receptors (TLRs), Toll-like receptor 4 (TLR4) plays a key role in recognizing and resisting the infection of bacteria in ducks [15]. LPS could be recognized and bound by TLR4, activating downstream inflammatory cascades of signal transduction ways, such as the nuclear factor kappa B (NF-κB) and Nod-like receptor families, and pyrin domain containing 3 (NLRP3) inflammasome [16,17]. The inhibition of the TLR4/NF-κB signaling pathway is an important method of alleviating LPS-induced inflammation [18]. Recent research has shown that there is an obvious relationship between oxidative stress and the expression of pro-inflammatory cytokines, such as interleukin-1β (IL-1β), interleukin-6 (IL-6), and tumor necrosis factor-α (TNF-α) [19]. Oxidative stress is an important mechanism of LPS-induced body injury. A few studies have demonstrated that LPS increases the production of reactive oxygen species (ROS) through different signaling pathways, thus resulting in oxidative stress [20]. Nuclear factor E2-related factor 2 (Nrf2) is a typical transcription factor that effectively regulates oxidative stress, thus having a protective effect on the tissue damage caused by the excessive production of ROS, which could alleviate a variety of diseases caused by LPS-induced oxidative stress [21,22].

Supplementation with RES in the diet exerted an anti-inflammatory effect by inhibiting the NF-κB activation induced by TLR4-mediated signaling [23]. Moreover, the downstream targets of NF-κB, which include IL-6 and interleukin-8 (IL-8), were downregulated significantly, which may be related to the anti-inflammatory ability of RES [24]. Rubiolo et al. (2008) reported that dietary RES supplementation promoted the nuclear translocation of Nrf2 and improved the expression levels of phase II detoxifying enzymes and other antioxidant enzymes [25]. Additionally, RES reduced malondialdehyde (MDA) content in tissue and alleviated oxidative stress in animal sepsis models through activating the Nrf-2 signaling pathway [26]. In recent years, crosstalk has been noted between Nrf2 and NF-κB [27,28]. Nrf2 and NF-κB signaling pathways may play important roles in the resistance of RES to LPS-induced inflammation. However, whether dietary RES can be of help in improving growth performance and reducing inflammatory response via Nrf2 and NF-κB signaling pathways need to be investigated.

It is important to study the relationship between inflammation and the growth performance of poultry to reduce economic losses in the poultry industry. In this study, LPS was used to establish a systemic inflammation duck model to study the effects of dietary RES on the systemic inflammation and growth performance of ducks. In particular, the levels of Nrf2 and NF-κB and their downstream targets in duck liver tissue were evaluated to further study the antagonistic effect of Nrf2 and NF-κB on the regulation of inflammatory reactions.

## 2. Materials and Methods

### 2.1. Chemicals

RES was purchased from Nanjing Nutri-herb Biotech Co., Ltd. (Nanjing, China, CAS: 501-36-0), and the purity was more than 98% by HPLC analysis. LPS (purity ≥ 98%, *Escherichia coli*055: B5) was acquired from (Sigma-Aldrich (St. Louis, MO, USA)), and was dissolved in phosphate-buffered solution (PBS) to obtain a 2.5 mg/mL LPS solution.

### 2.2. Ethics Approval

The animal experimental procedures were approved by the Ethical and Animal Welfare Committee of China’s Heilongjiang Province. The ducks were fed in the Experimental Base of Northeast Agricultural University. All the ducks were allowed ad libitum access to water and feed during the experimental period. The experimental protocol of this study was subjected to approval by the Northeast Agricultural University Institutional Animal Care and Use Committee (Protocol number: NEAU [2011]-9).

### 2.3. Ducks and Husbandry

A total of 280 one-day-old SPF male ducks (body weight of 35 ± 1 g *Anas platyrhynchos*) from the same incubator were caged (4 per cage) and randomly divided into 2 dietary treatments for the growth performance experiment: R_0_, corn-soybean basal diet; R_400_, corn-soybean basal diet + 400 mg kg^−1^ RES. The basal diets were in accordance with the National Research Council (1994) (Table S1). At the age of 28 days, 16 ducks were selected from each treatment group and divided into four subgroups (8 ducks per group) for a 2 × 2 factorial pathological experiment: R_0_, corn-soybean basal diet injected with 2 mL PBS/kg body weight; R_400_, corn-soybean basal diet + 400 mg kg^−1^ RES injected with 2 mL PBS/kg body weight; R_0_ + LPS, corn-soybean basal diet injected with 5 mg LPS/kg body weight; R_400_ + LPS, corn-soybean basal diet + 400 mg kg^−1^ RES injected with 5 mg LPS/kg body weight (Table 1). After 12 h of fasting, 8 ducks per subgroup were slaughtered for sample collection.

### 2.4. Sample Collection

Blood samples (10 mL) were obtained from the wing vein of ducks into anticoagulation tubes containing heparin. The plasma was separated by centrifugation for 5 min at 1000× *g* at 4 °C and stored at −80 °C until used. The liver samples were collected and fixed with 4% paraformaldehyde solution and 2.5% glutaraldehyde, respectively. The remaining liver tissue was stored at −80 °C for further analysis of enzyme activity, quantitative real-time PCR, and Western blot.

### 2.5. Histopathological Analysis of Liver

The liver sample fixed in 4% paraformaldehyde was processed through transparent dehydration with wax and sliced into microtome sections of 5 μm thickness. Sections were then deparaffinized, rehydrated, and stained with hematoxylin-eosin. After scanning the tissue section with panoramic MIDI (3D Histech, Budapest, Hungary), the histological morphometric variables were analyzed, quantified, and photographed using virtual microscope software (Image-Pro Plus 6.0, 3D Histech) [8]. Eight visual fields of each sample were randomly selected and observed.

### 2.6. Transmission Electron Microscopy (TEM)

Liver samples were cut into small pieces and fixed with 2.5% glutaraldehyde. Electron microscopy sample preparation was conducted as previously reported [8]. After being dried with CO_2_ in a Hitachi Model HCP-2 critical point dryer, the samples were covered with gold-palladium. One sample and one backup sample were prepared for each duck. Fifteen visual fields of each sample were randomly selected and observed under a digital TEM (Hitachi S-4800, Tokyo, Japan).

### 2.7. Assay of the Antioxidant Levels of the Plasma and the Liver

The total liver sample (0.10 g) was weighed and added to 0.9 mL of stroke-physiological saline solution (SPSS, 4 °C, 0.9% NaCl, pH = 7.2–7.4). Then, the 10% liver/SPSS homogenate was obtained with a low-temperature, high-speed grinder (LAWSON-24, LAWSON, Beijing, China) at 4 °C. The homogenate was centrifuged, and the supernatant was collected. Antioxidant biomarkers of the supernatant were determined by assay kits (Nanjing Jiancheng Bioengineering Institute, Nanjing, China) with a UV-VIS spectrophotometer (UV1100, MAPADA, Shanghai, China). The assay kits used in this experiment were as follows: T-SOD assay kit (catalog number: A001-1-2a), GSH-Px assay kit (catalog number: A005-1-2), GSH-ST assay kit (catalog number: A004-1-1), and MDA assay kit (catalog number: A003-1-2).

### 2.8. Quantitative Real-Time PCR (qRT-PCR)

Total RNA from each sample of the duck liver (100 mg) was isolated using a reagent kit (catalog number: 9108, TaKaRa, Tokyo, Japan) as recommended by the manufacturer. The concentration and purity of total RNA were examined by the A260/A280 ratio with a spectrophotometer (Implen Nanophotometer P-330, Munich, Germany). One μg of total RNA from each sample of duck liver was transformed into cDNA with a Prime Script™ RT reagent kit with gDNA Eraser (catalog number: RR047A, TaKaRa, Dalian, China) according to the protocol recommended by manufacturers. The qRT-PCR was performed with 1 μL of obtained first-strand cDNA from each liver sample with a template for a TB Green™ Premix Ex Taq™ (catalog number: RR086A, TaKaRa, Dalian, China) RT-PCR (qRT-PCR) kit. The gene accession numbers of the duck were gained from NCBI, and the duck gene primers were purchased from Sangon Biotech Co., Ltd. (Shanghai, China) (Table S2). All PCR assays were determined on the same 96-hole PCR plate with two repetitions. The RT-PCR condition was run in the PCR System (ABI 7500, New York City, NY, USA): one cycle at 95 °C for 30 s, 40 cycles at 95 °C for 5 s, and at 60 °C for 30 s. The relative gene expression ratio of the target mRNA was detected using the 2^−ΔΔCt^ method and normalized to β-actin expression.

### 2.9. Western Blotting

Western blotting was conducted as previously described [8]. Similarly, we obtained the supernatant of the sample and extracted proteins using the radio immunoprecipitation assay (RIPA) buffer containing 1 mmol/L PMSF (Beyotime, Shanghai, China), and the protein concentration was quantified using a BCA assay kit (Beyotime, Shanghai, China). During the process of electrophoresis, the proteins with different sizes were separated in SDS-PAGE. Then, the proteins on the gel were transferred onto a polyvinylidene-difluoride (PVDF) membrane (Beyotime, Shanghai, China) for blotting. Antibodies were acquired from Beyotime Biotechnology, Shanghai, China, which included glyceraldehyde-3-phosphate dehydrogenase (GAPDH) mouse monoclonal antibody (catalog number: AG019), caspase-1rabbit polyclonal antibody (catalog number: AF1681), Nrf2 rabbit polyclonal antibody (catalog number: AF7623), NF-κB p65 rabbit polyclonal antibody (catalog number: AF5875), HO-1rabbit polyclonal antibody (catalog number: AF1333), NLRP3 rabbit monoclonal antibody (catalog number: AF2155), HRP-labeled goat anti-rabbit IgG (H + L) (catalog number: A0208) and HRP-labeled goat anti-mouse IgG (H + L) (catalog number: A0216). Original Western Blot figures in Figure S2.

### 2.10. Statistical Analysis

The experimental data of each sample were obtained from eight measurements. Results are expressed as mean ± standard deviation (mean ± SD) and analyzed using SPSS (version 22.0, SPSS Inc., Chicago, IL, USA). Statistical significance of the date was evaluated using ANOVA followed by a least significant difference (LSD) test as the post-hoc test with a 5% probability of error and a value of *p* < 0.05 was considered statistically significant. All the graphs with standard deviation bar were made by GraphPad Prism in this study (version 8.3.0, GraphPad Software, San Diego, CA, USA).

## 3. Results

### 3.1. Effect of Dietary RES on the Growth Performance in Ducks

After 28 days of treatment with dietary RES, the final weight of the R_400_ group was significantly higher than that of the R_0_ group (*p* < 0.05). Furthermore, dietary RES showed a significantly positive effect on increasing the ADG of ducks (*p* < 0.05) (Table 2).

### 3.2. Effect of Dietary RES on the Plasma Biochemistry in Ducks

The results showed that there was no difference in the plasma levels of TP (total protein), ALP (alkaline phosphatase), or GLOB (globulin) among the four groups. The R_0_ + LPS group showed markedly reduced plasma levels of ALB (albumin) and A/G (albumin/globulin), compared to the ducks in the R_0_ group (*p* < 0.05). These reductions were significantly alleviated by dietary RES (*p* < 0.05). Compared with the R_0_ group, the plasma levels of ALT (cereal third transaminase), AST (aspartate aminotransferase), Cr, TB (total bilirubin), and BUN (blood urea nitrogen) were significantly increased in the R_0_ + LPS group (*p* < 0.05). However, the plasma levels of ALT, AST, Cr, TB, and BUN in the R_400_ + LPS group were higher than those in the R_0_-LPS group (*p* < 0.05). In addition, the plasma level of ALT in the R_400_ group was lower than that in the R_0_ group (*p* < 0.05) (Table 3).

### 3.3. Effect of Dietary RES on the Expression of Inflammatory Cytokines in Systemic and Duck Liver

Among the four groups, the plasma levels of IL-1β, IL-6, and TNF-α in the R_0_ + LPS group were significantly higher than the other three groups (*p* < 0.05). Compared to the R_0_ group, the plasma levels of IL-1β, IL-6, and TNF-α were significantly decreased in the R_400_ group (*p* < 0.05). The plasma levels of IL-1β, IL-6, and TNF-α in the R_400_ + LPS group were significantly lower than those in the R_0_ + LPS group (*p* < 0.05). In addition, no significant difference in the plasma levels of IL-1β, IL-6, or TNF-α were found between the R_0_ group and the R_400_ + LPS group. As shown in Table 4, the liver protein levels of IL-1β, IL-6, and TNF-α were highest in the R_0_ + LPS group (*p* < 0.05). Given the results of the analysis, the liver protein levels of IL-1β, IL-6, and TNF-α in the R_400_ group were significantly lower than those of the R_0_ group (*p* < 0.05). However, the content of IL-6 and TNF-α in the liver of LPS-induced ducks treated with 400 mg/kg dietary RES was lower than those of LPS-induced ducks in the R_0_ + LPS group (*p* < 0.05). Moreover, no significant difference in the liver levels of IL-1β and TNF-α was found between the R_0_ group and the R_400_ + LPS group. However, the liver level of IL-6 in the R_400_ + LPS group was significantly decreased compared to the R_0_ group (Table 4).

### 3.4. Effect of Dietary RES on Regulation of TLR4/NF-κB Signaling Pathway

**Figure 1:**
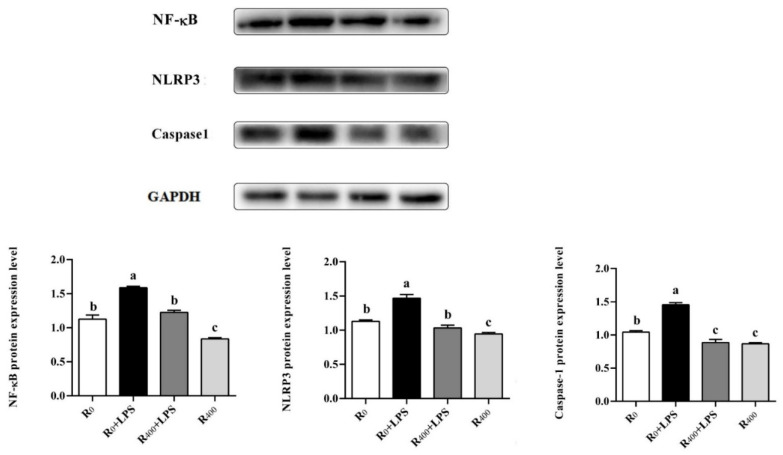
Effect of RES on the protein expression of NF-κB, NLRP3, and caspase-1 in the duck liver. Values are expressed as mean ± SEM. Labeled (a, b, c, a > b > c) means in a row without a common letter differ, *p* < 0.05. Original Western Blot figures in Figure S2.

The mRNA levels of TLR4, NF-κB, p53, and NLRP3 in the R_0_ + LPS group were significantly higher than the other three groups (*p* < 0.05). No significant difference in the mRNA levels of TLR4, NF-κB, p53, and NLRP3 was found between the three groups. Compared with the R_0_ group, the mRNA levels of TXNIP, caspase-1, IL-6, and TNF-αwere significantly decreased in the R_400_ group (*p* < 0.05) and significantly increased in the R_0_ + LPS group (*p* < 0.05). In addition, no significant differences were noted between the R_0_ group and the R_400_ + LPS group (Table 5). Compared with the R_0_ group, LPS challenge significantly increased the protein levels of NF-κB, NLRP3, and caspase-1 in the R_0_ + LPS group (*p* < 0.05). Compared with the R_0_ group, the protein levels of NF-κB, NLRP3 and caspase-1 were significantly decreased in the R_400_ group (*p* < 0.05). There were no statistical differences in the protein levels of NF-κB and NLRP3 between the R_0_ group and R_400_ + LPS group. However, the protein level of caspase-1 was lower in the R_400_ + LPS group compared with the R_0_ group (*p* < 0.05). In addition, the protein level of caspase-1 was no different between the R_400_ + LPS group and the R_400_ group (Figure 1).

### 3.5. Effect of Dietary RES on Regulation of Nrf2/HO-1 Signaling Pathway and Anti-Oxidase Activity in Duck

**Figure 2:**
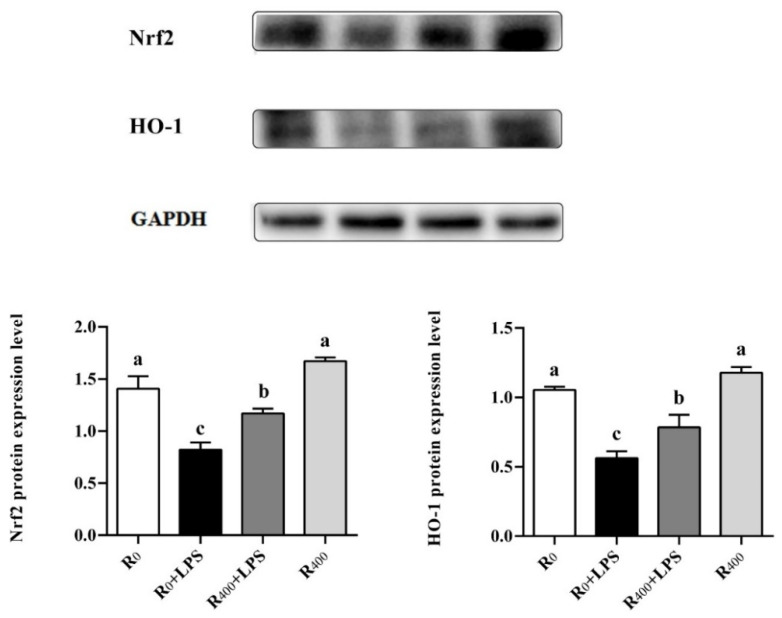
Effect of RES on the protein expression of Nrf2 and HO-1 in the liver of LPS-induced duck. Values are expressed as mean ± SEM. Labeled (a, b, c, a > b > c) means in a row without a common letter differ, *p* < 0.05. Original Western Blot figures in Figure S2.

The mRNA and protein levels of Nrf2 in the R_0_ + LPS group were significantly lower than those in the R_0_ group and markedly lower than those in the R_400_ + LPS group (*p* < 0.05). Compared with the R_0_ group and R_400_ group, dietary RES significantly increased the mRNA expression of Nrf2 (*p* < 0.05) but had no significant effect on the protein expression of Nrf2. Compared to the mRNA and protein levels of HO-1 in the R_0_ group, the levels were significantly increased in the R_400_ group (*p* < 0.05) (Figure 2). According to the result, the gene expression of HO-1 was increased by supplementing with 400 mg/kg RES in LPS-induced ducks (*p* < 0.05). The protein expression of HO-1 in the R_0_ + LPS group was significantly decreased compared to that in the R_0_ group (*p* < 0.05). LPS challenge affected the expression of Keap1 so that the gene level of Keap1 in the R_0_ + LPS group was higher than that of the R_0_ group (*p* < 0.05). The gene level of superoxide dismutase (SOD) in the R_0_ group was higher than that in the R_0_ + LPS group (*p* < 0.05) and lower than that in the R_400_ group (*p* < 0.05). The mRNA expression of glutamate-cysteine ligase catalytic (GCLC) (*p* < 0.05) and glutamate-cysteine ligase modifier subunit (GCLM) (*p* < 0.05) in the R_0_ + LPS group markedly lower than that in the R_400_ + LPS group. Meanwhile, the mRNA expression of GCLM in the R_0_ + LPS was lower than that in the R_0_ group (Table 6). The results of this study showed that compared with the R_0_ group, the activities of liver antioxidant enzymes, including T-SOD and GSH-Px, were significantly decreased by LPS stimulation in the R_0_ + LPS group (*p* < 0.05). However, there was a significant improvement of T-SOD and GSH-Px activities between the R_400_ + LPS group and the R_0_ + LPS group. Notably, the activity of GSH-ST showed different trends, which, by the activity of GSH-ST in the R_0_ + LPS group, was higher than that in the R_0_ group (*p* < 0.05) and lower than that in the R_400_ + LPS group (*p* < 0.05). In addition, the MDA level in the livers of the R_0_ + LPS group were higher than that in the R_0_ group (*p* < 0.05) and the R_400_ + LPS group (*p* < 0.05), and the MDA level in the livers of the R_0_ group was higher than that in the R_400_ group (*p* < 0.05) (Table 7).

## 4. Discussion

As a high-quality alkaline protein source, duck meat comprises an important part of the poultry market [29]. At present, infection by multiple pathogens is a common and severe challenge for the meat production system [30]. Plant polyphenols, as the secondary metabolites of natural plants, exist widely in most natural plants. They usually have antioxidant and anti-inflammatory properties due to their special molecular structure [31]. RES is considered a typical plant polyphenol with many physiological effects, such as prevention of various diseases, weight control, and anti-inflammatory and antioxidant activities [32,33]. The present study is the first to demonstrate that dietary RES improves the feeding performance of ducks, which might be correlated with its anti-inflammatory or antioxidant benefits.

As a result of this study, higher final BW and ADG were observed in those ducks fed RES. During the last decade, various studies have corroborated that RES used as a feed additive can cause a positive effect on growth performance. Wang et al. (2021) reported that 400 mg/kg of RES improved the growth performance of the final BW and ADG in heat-stressed Arbor Acres broilers [34]. Similarly, a dietary combination of RES and curcumin at a high dose (300 mg/kg) improved the ADG, ADFI, and FCR and had a marked effect on the apparent digestibility of nutrients in pigs. On the contrary, the dietary combination of RES and curcumin at a low dose (100 mg/kg) had no effect on the growth performance of piglets. This may be due to the low bioavailability of polyphenolics [35]. Based on the previous studies, the high concentration of dietary RES (400 mg/kg) was chosen to ensure the effective promotion of the growth performance of ducks in this study.

It has previously been reported that inflammation is significantly linked to higher metabolic expenditure [36]. As a mainly pathogenic component of Gram-negative bacteria, LPS was used in many studies to establish inflammatory animal models to explore various inflammatory responses due to the characteristic that LPS could simulate bacterial infection without the risk of infection caused by living bacteria [37]. To further explore the relationship between inflammation and the reduction in growth performance, two other experimental groups were created with LPS treatment. Clinically, ALB was regarded as an acute-phase protein that triggers the acute systemic inflammatory response induced by bacterial infection, which is accompanied by hypoproteinemia and could translate into increased mortality [38]. Biochemical analysis of the plasma samples in this study showed that there were marked reductions in ALB and A/G plasma levels induced by LPS compared to the ducks in the R_0_ group, and these reductions were significantly alleviated by dietary RES. This suggests that dietary RES could attenuate hypoproteinemia and improve the overall state of the organism. Moreover, the inflammatory response induced by LPS could cause organ injury and subsequent release of pro-inflammatory markers, which include BUN, ALT, and AST [39,40]^.^ Several studies have previously shown that RES was recognized as a suppressor of the inflammatory response that can attenuate hepatocyte injury and kidney mitochondrial dysfunction to protect the functions of multiple organs [41]. Similarly, the plasma levels of ALT, AST, and BUN were obviously increased by LPS treatment in the R_0_ + LPS group, and dietary RES induced a significant decrease in ALT, AST, and BUN plasma levels in R_400_ + LPS group in this study. These results reveal that dietary RES played a protective role in LPS-induced inflammation and reduced organ damage in ducks.

To examine the protective effects of RES on the LPS-induced inflammatory pathways, the levels of various inflammatory cytokines (IL-1β, IL-6, TNF-α) in plasma and liver were assessed. Consistent with previous research, LPS challenge increased the levels of IL-1β, IL-6, and TNF-α in the R_0_ + LPS group [10]. Our results show that plasma levels of IL-1β and TNF-α in the R_400_ group were significantly lower than those in the R_0_ group and that plasma levels of IL-1β and IL-6 in the R_400_ + LPS group were significantly lower than those in the R_0_ + LPS group. These results suggest that dietary RES reduces systemic inflammation and hepatic inflammation in healthy ducks as well as in LPS challenge ducks. Previous research has suggested that LPS challenge resulted in a liver inflammatory response and induced pathological changes in liver cells that included the shrinkage of the nucleus and the dilatation of the rough endoplasmic reticulum. However, RES and pterostilbene (A dimethyl ether analog of RES) could alleviate liver injury and endoplasmic reticulum stress induced by early weaning in piglets [42]. In this study, the ultrastructure of duck liver was evaluated, and the results are consistent with previous studies (Figure S1A). Furthermore, as a result of HE staining, LPS induced hepatocellular vacuolation, interstitial bleeding, and inflammatory cell infiltration (Figure S1B). These results provide evidence that RES can alleviate the duck liver injury caused by LPS.

To further uncover the molecular mechanism of dietary RES’s protective effects on growth performance and LPS-induced inflammation, Western blot and real-time quantitative PCR analysis were performed. Pattern recognition receptors (PRRs) are regarded as a repertoire of germline-encoded proteins related to the innate immune system. Toll-like receptors (TLRs) are an important class of PRRs, that can effectively and widely recognize many pathogens. As a cellular receptor family of pathogens, TLRs can regulate the inflammatory response induced by microbial challenges in vertebrates. Previous studies have provided evidence that the mRNA expression level of TLR4 in the intestinal tissue of chicken embryos was upregulated by LPS treatment [43]. Extracellular LPS can be recognized or absorbed by the cell and leads to the activation of the NF-κB signaling pathway followed by the secretion of pro-inflammatory cytokines and inflammation [44]. In order to confirm the effect of the TLR4/NF-κB signaling pathway on the regulation of inflammatory response in ducks, TLR4, NF-κB, and p53 mRNA levels and NF-κB protein level in the liver were measured in this study. Our results showed that dietary RES inhibited the gene expression of TLR4, NF-κB, and p53 and the protein expression of NF-κB both in healthy ducks and LPS challenge ducks, which was consistent with previous reports that RES suppressed the TLR4 signaling pathway in the spleen of yellow-feather broilers [45]. Additionally, NLRP3 is an important inflammasome in animals and humans, which can be activated by TXNIP binding and recognize various pathogens of the immune system [46]. The activation of NLRP3 led to an increase in caspase-1 protein level and played an important role in the secretion of pro-inflammatory cytokines associated with inflammatory response [47]. In this study, dietary RES significantly reduced IL-6 and TNF-α mRNA levels, and the mRNA expression of TXNIP, NLRP3, and caspase-1, and the protein expression of NLRP3 and caspase-1 was also reduced significantly in the liver of both healthy ducks and LPS challenge ducks. Our results showed that the TLR4/NF-κB signaling pathway in duck liver was activated by LPS and triggered the activation of a downstream inflammatory network that included the TXNIP/NLRP3 and caspase-1 pathways. These results were consistent with previous in vitro research in ducks [15]. However, dietary RES inhibited the activation of the TLR4/NF-κB signaling pathway and significantly reduced the mRNA and protein levels of inflammatory cytokines. Similar results have been reported where RES could induce the inhibition of NLRP3 and caspase-1 in heat-stressed ducks [10]. These results demonstrated that dietary RES alleviated the LPS-induced inflammatory response in ducks by inhibiting the activation of the TLR4/NF-κB and TXNIP/NLRP3 signal pathways.

In recent years, oxidative stress has been shown to increase systemic inflammation, and a close connection between LPS-induced systemic inflammation and oxidative stress has been reported [48,49]. Previous research has revealed that the Nrf2/HO-1 pathway plays be an essential role in the molecular mechanism of endogenous antioxidant stress [50]. As the principal negative regulator of the Nrf2 signal pathway, Kelch-like ECH-associated protein 1 (Keap1) plays a central role in the balance of intracellular redox homeostasis [51]. Nrf2 has been reported to be associated with the activation of multiple antioxidation enzymes, including SOD, catalase (CAT), HO-1, NQO1, GCLC, and GCLM [52,53]. Yang et al. (2019) reported that Nrf2 was considered to be a key target gene for the effects of RES on the upregulation of antioxidant genes and improvement of antioxidant capacity in ducks [11]. In this study, the single administration of LPS-induced oxidative stress and the inhibition of the Nrf2/HO-1 signaling pathway, resulting in a reduction in the protein level of Nrf2 and HO-1. In addition, LPS challenge also downregulated the expression of Nrf2-downstream antioxidant genes, which include SOD and GCLM, and the activities of antioxidant enzymes (GSH-Px, GSH-ST, and T-SOD) were decreased. In contrast, pretreatment with dietary RES significantly diminished the mRNA expression of Nrf2 and HO-1 and improved the antioxidant capacity of ducks. Importantly, in healthy ducks without the administration of LPS, dietary RES also upregulated the Nrf2 and HO-1 protein level and reduced the production of MDA. This suggests that dietary RES improves the antioxidant capacity and alleviates oxidative stress during the raising of ducks. This underlying mechanism is consistent with our previous studies that found that RES reinforces antioxidative activities to reduce water loss and improve duck meat quality [9].

It is was generally accepted that oxidative stress and inflammation are interconnected pathophysiological processes associated with many kinds of inflammatory diseases [54] and that the overproduction of inflammatory mediators could be used to determine oxidative stress. There is a connection between the suppression of the NF-κB signaling pathway and the activation of the Nrf2 signaling pathway, while Nrf2 overexpression suppresses NF-κB-DNA binding activity [55]. Various plant extracts have been reported that could induce the Nrf2 expression and change the cellular redox status to regulate the inactivation of NF-κB [56]. In all groups in this study, the activation of Nrf2 had a significant tendency to inhibit the expression of NF-κB. Our results suggest that dietary RES activates the Nrf2 signaling pathway and regulates the inhibition of the NF-κB signaling pathway, possibly protecting against inflammation and oxidative stress in ducks. Furthermore, dietary RES may reduce the high rate of catabolism caused by inflammatory responses leading to a significant improvement in the growth performance of ducks.

## 5. Conclusions

In conclusion, the results of this study yield an attractive molecular mechanism by which dietary RES could reduce oxidative stress and inflammation through the activation of the Nrf2/HO-1 signaling pathway and the inactivation of the TLR4/NF-κB signaling pathway. In this study, the antioxidant capacity of ducks was improved by dietary RES, which increased antioxidant enzyme levels and reduced MDA production in both healthy and LPS-induced duck models. In addition, dietary RES effectively attenuated LPS-induced hepatitis and liver damage. Moreover, dietary RES alleviated the systemic inflammatory response and reduced the variation in blood biochemistry levels after LPS challenge in ducks. Based on the above findings, a possible mechanism is proposed by which dietary RES improved duck growth performance via its antioxidant and anti-inflammatory properties.

## Figures and Tables

**Table 1:** Experimental groups and treatment.

Experimental Groups	Basal Diet	RES (mg/kg Basal Diet)	Number Ducks per Treatment Diet
Growth performance experiment R_0_	Corn-soybean	0	140
R_400_	Corn-soybean	400	140
Pathological experiment			
R_0_	Corn-soybean	0	8
R_0_ + LPS	Corn-soybean	0	8
R_400_ + LPS	Corn-soybean	400	8
R_400_	Corn-soybean	400	8

Note: RES: Dietary RES; R_0_: the group fed with the corn-soybean basal diet; R_400_: the group fed with the corn-soybean basal diet and supplemented with 400 mg/kg dietary RES; R_0_ + LPS: the group fed with the corn-soybean basal diet and injected with 5 mg LPS/kg body weight; R_400_ + LPS: the group fed with the corn-soybean basal diet and supplemented with 400 mg/kg dietary RES and injected with 5 mg LPS/kg body weight.

**Table 2:** Effect of dietary RES on growth performance in ducks.

Items	Groups		SEM	*p*-Value
	R_0_	R_400_		
Initial BW (g)	35.13 ± 0.41	35.12 ± 0.36	0.37	0.754
Final BW (g)	647.83 ± 20.20	661.43 ± 15.09	9.41	0.005
ADFI (g/day)	61.08 ± 1.89	59.61 ± 1.63	1.69	0.727
ADG (g/day)	21.88 ± 0.27	22.37 ± 0.19	0.33	0.005
F/G (g/g)	2.79 ± 0.10	2.66 ± 0.08	0.97	0.182

Note: The values are shown as mean ± SD of 140 individual male ducks. SEM: total standard error; *p* < 0.05 represents a significant regression relationship; *p* is the comparison value of the two groups. ADG: average weight gain, ADFI: average daily feed intake, F/G: the ratio of feed to gain.

**Table 3:** Effect of dietary RES on plasma biochemistry in health ducks and LPS-challenged ducks.

Items	Groups			
	R_0_	R_0_ + LPS	R_400_ + LPS	R_400_
TP (g/L)	30.60 ± 4.45	29.40 ± 4.61	33.60 ± 4.60	31.40 ± 2.19
ALB (g/L)	21.04 ± 0.66 ^a^	17.52 ± 0.85 ^b^	19.56 ± 2.18 ^a^	21.12 ± 1.87 ^a^
GLOB (g/L)	13.02 ± 1.32	12.38 ± 1.20	14.74 ± 1.89	13.66 ± 1.43
A/G	1.46 ± 0.08 ^a^	1.19 ± 0.09 ^c^	1.34 ± 0.10 ^b^	1.49 ± 0.09 ^a^
TB (μmol/L)	1.14 ± 0.24 ^b^	1.66 ± 0.21 ^a^	1.32 ± 0.21 ^b^	1.17 ± 0.13 ^b^
ALP (KDa)	478.8 ± 31.2	470.2 ± 32.37	525.4 ± 48.39	503.4 ± 41.03
ALT (U/L)	32.80 ± 0.98 ^b^	40.00 ± 6.06 ^a^	34.40 ± 2.42 ^b^	30.60 ± 2.49 ^c^
AST (U/L)	31.50 ± 0.98 ^b^	40.80 ± 3.44 ^a^	34.00 ± 2.44 ^b^	31.80 ± 4.78 ^b^
Cr (μmol/L)	22.00 ± 4.05 ^b^	28.60 ± 4.03 ^a^	23.60 ± 2.06 ^b^	20.06 ± 3.92 ^b^
BUN (mmol/L)	0.18 ± 0.07 ^b^	0.40 ± 0.06 ^a^	0.24 ± 0.05 ^b^	0.22 ± 0.04 ^b^

Note: The values are shown as mean ± SD of 8 individual male ducks. TP: total protein; ALB: albumin; GLOB: globulin; A/G: albumin/globulin; TB: total bilirubin; ALP: alkaline phosphatase; ALT: cereal third transaminase; AST: aspartate aminotransferase; Cr: creatinine; BUN: blood urea nitrogen. Labeled (a, b, c, a > b > c) means in a row without a common letter differ, *p* < 0.05.

**Table 4:** Effect of dietary RES on the plasma and liver tissue level of inflammatory cytokines (IL-1β, IL-6, and TNF-α) in LPS-challenged ducks.

Items	Groups			
	R_0_	R_0_ + LPS	R_400_ + LPS	R_400_
The serum contents of inflammatory factors				
IL-1β (pg/mL)	23.51 ± 3.12 ^b^	31.14 ± 0.73 ^a^	21.08 ± 1.47 ^b^	17.12 ± 1.03 ^c^
IL-6 (pg/mL)	123.24 ± 7.21 ^b^	159.65 ± 4.22 ^a^	128.94 ± 6.85 ^b^	115.10 ± 8.39 ^c^
TNF-α (pg/mL)	63.08 ± 9.73 ^b^	76.35 ± 5.01 ^a^	59.27 ± 3.19 ^b^	50.36 ± 3.69 ^c^
The liver contents of inflammatory factors				
IL-1β (pg/mg protein)	2.26 ± 0.17 ^b^	2.96 ± 0.37 ^a^	2.58 ± 0.58 ^b^	1.83 ± 0.32 ^c^
IL-6 (pg/mg protein)	7.38 ± 0.31 ^c^	8.96 ± 0.29 ^a^	8.18 ± 0.48 ^b^	5.69 ± 0.45 ^d^
TNF-α (pg/mg protein)	61.84 ± 4.05 ^b^	69.18 ± 4.65 ^a^	65.38 ± 2.56 ^b^	50.36 ± 3.69 ^c^

Note: The values are shown as mean ± SD of 8 individual male ducks. Labeled (a, b, c, d, a > b > c > d) means in a row without a common letter differ, *p* < 0.05.

**Table 5:** Effect of RES on the mRNA level of the anti-inflammatory genes in LPS-challenged ducks.

Items	Groups			
	R_0_	R_0_ + LPS	R_400_ + LPS	R_400_
TLR4	1.01 ± 0.16 ^b^	1.63 ± 0.51 ^a^	0.85 ± 0.12 ^b^	0.79 ± 0.13 ^b^
NF-κB	1.00 ± 0.04 ^b^	1.78 ± 0.50 ^a^	1.09 ± 0.11 ^b^	0.75 ± 0.18 ^b^
p53	1.01 ± 0.18 ^b^	3.06 ± 1.07 ^a^	1.20 ± 0.20 ^b^	0.85 ± 0.25 ^b^
NLRP3	1.06 ± 0.13 ^b^	1.70 ± 0.23 ^a^	1.10 ± 0.40 ^b^	0.85 ± 0.08 ^b^
TXNIP	1.01 ± 0.14 ^b^	1.43 ± 0.38 ^a^	0.81 ± 0.13 ^b^	0.68 ± 0.16 ^c^
Caspase-1	1.00 ± 0.09 ^b^	1.31 ± 0.37 ^a^	0.92 ± 0.14 ^b^	0.65 ± 0.12 ^c^
IL-6	1.02 ± 0.23 ^b^	1.57 ± 0.40 ^a^	0.83 ± 0.17 ^b^	0.63 ± 0.13 ^c^
TNF-α	1.01 ± 0.11 ^b^	1.88 ± 0.67 ^a^	1.12 ± 0.14 ^b^	0.59 ± 0.19 ^c^

Note: The values are shown as mean ± SD of 8 individual male ducks. Labeled (a, b, c, a > b > c) means in a row without a common letter differ, *p* < 0.05.

**Table 6:** Effect of RES on the mRNA level of the antioxidant genes in LPS-challenged ducks.

Items	Groups			
	R_0_	R_0_ + LPS	R_400_ + LPS	R_400_
Nrf2	1.00 ± 0.10 ^b^	0.70 ± 0.16 ^c^	1.02 ± 0.10 ^b^	1.48 ± 0.26 ^a^
Keap1	1.02 ± 0.23 ^b^	1.65 ± 0.32 ^a^	1.28 ± 0.41 ^b^	0.95 ± 0.16 ^b^
HO-1	1.00 ± 0.08 ^b^	0.86 ± 0.17 ^c^	1.24 ± 0.33 ^b^	1.86 ± 0.52 ^a^
SOD1	1.05 ± 0.20 ^b^	0.63 ± 0.16 ^b^	0.86 ± 0.28 ^b^	1.92 ± 0.52 ^a^
GCLC	1.01 ± 0.19 ^b^	0.89 ± 0.26 ^b^	1.28 ± 0.23 ^a^	1.26 ± 0.26 ^a^
GCLM	1.01 ± 0.11 ^b^	0.78 ± 0.16 ^c^	1.16 ± 0.18 ^a^	1.33 ± 0.31 ^a^

Note: The values are shown as mean ± SD of 8 individual male ducks. Labeled (a, b, c, a > b > c) means in a row without a common letter differ, *p* < 0.05.

**Table 7:** Effect of RES on the plasma level of the antioxidant capacity in ducks.

Items	Groups			
	R_0_	R_0_ + LPS	R_400_ + LPS	R_400_
GST (U/mg protein)	18.14 ± 1.46 ^c^	23.61 ± 1.40 ^a^	20.11 ± 2.02 ^b^	16.91 ± 0.82 ^c^
GSH-PX (U/mg protein)	383.38 ± 27.40 ^b^	311.63 ± 51.87 ^c^	342.97 ± 33.51 ^b^	422.38 ± 54.62 ^a^
T-SOD (U/mg protein)	375.53 ± 39.38 ^a^	330.26 ± 11.94 ^c^	302.03 ± 22.77 ^b^	381.01 ± 43.64 ^a^
MDA (nmol/mg)	1.06 ± 0.14 ^c^	1.40 ± 0.05 ^a^	1.44 ± 0.10 ^b^	1.12 ± 0.34 ^d^

Note: The values are shown as mean ± SD of 8 individual male ducks. T-SOD: total superoxide dismutase; GSH-Px: glutathione peroxidase; GST: glutathione s-transferase; MDA: malondialdehyde; Labeled (a, b, c, d, a > b > c > d) means in a row without a common letter differ, *p* < 0.05.

## Data Availability

Not available.

## Supplementary Materials

The following are available online at https://www.mdpi.com/article/10.3390/ani11123588/s1, Figure S1: Effect of RES on the liver morphology and inflammatory response in duck ileum challenged by LPS, Figure S2: Original Western Blot figures, Table S1: Ingredients and nutrient composition of basal diet (on an air-dried basis), Table S2: Primer sequences and productlengths of target genefragments.
