# Supplementary material for: Effects of Dietary Resveratrol Supplementation on Growth Performance and Anti-Inflammatory Ability in Ducks (Anas platyrhynchos) through the Nrf2/HO-1 and TLR4/NF-κB Signaling Pathways
Source: Animals (Basel). 2021 Dec 18;11(12):3588. doi: 10.3390/ani11123588 (PMC8698092; doi:10.3390/ani11123588)
Supplement: Supplementary file 1 [file animals-11-03588-s001.zip › animals-1431513-supplementary.pdf]

Table S1. Ingredients and nutrient composition of basal diet (on an air-dried basis).

**Items**	%
Ingredient	
Corn (7.9)	61.70
Soybean meal (45)	26.09
Corn protein flour (55)	7.90
Dicalcium phosphate	1.40
Limestone	1.08
Sodium chloride	0.38
DL-Methionine	0.15
L-Lysine	0.20
Choline chloride (50%)	0.10
Premix	1.001
Total	100
Nutritional level	
Calculated nutrient	
Net energy (MJ/kg)	12.14
CP (%)	20.67
Calcium (%)	0.90
Total phosphorus (%)	0.68
Non-phytate phosphorus (%)	0.44
Items(%)	1–4 weeks
Methionine (%)	0.48
Methionine +cystine (%)	0.81
Threonine (%)	0.75
Tryptophane (%)	0.21

Note: Premix provided per kg of diet: Cu, 8 mg; Fe, 65 mg; Zn, 80 mg; Mn, 105 mg; I, 1 mg; Se, 0.3 mg; vitamin A, 9800 IU; vitamin D3, 3100IU; vitamin E, 26 IU; vitamin B1, 2.5 mg; vitamin B2, 7 mg; vitamin B12, 0.018 mg; vitamin K, 2.2 mg; biotin, 0.09 mg; folic acid, 1 mg; pantothenic acid, 11 mg; nicotinic acid, 38 mg.

Table S2. Primer sequences and productlengths of target genefragments.

Transcripts	Accession number		Primer sequence (5′-3′)	Product Length (bp)
Keap1	MF774811.1	Forward	TCACCCTCCATAAACCCACCCAAG	102
		Reverse	AGTAGCCCAAGGACTGCCGATAG	102
Nrf2	NM_001310777.1	Forward	GTTGAATCATCTGCCTGTGG	171
		Reverse	TAAGCTAGGTGGTCGAGTGC	172
HO-1	KU048806.1	Forward	AAGAGCCAGGAGAACGGTCACC	139
		Reverse	TGCCCACCAGGTCTGTCTGAC	139
SOD	XM_013097859.1	Forward	CCTGTGGTGTCATCGGAATA	116
		Reverse	TTGAACGAGGAAGAGCAAGTA	127
GCLC	XM_027455104.1	Forward	TTCAGGTGACATTCCAGGCTTGC	108
		Reverse	AGAACGGAGATGCAGCACTCAATG	108
GCLM	XM_027462629.1	Forward	TGTTGTGTGATGCCACCTGATCTC	150
		Reverse	CCATTCGTGTGCTTTGACGTTCTG	150
IL-6	JQ728554.1	Forward	ATGTGCGAGAAGTTCACCGTCTG	113
		Reverse	TCGTCGAAGCCAGCCAGGAG	113
TNF-α	XM_013105371.3	Forward	TCAGCTGGCTAAGACCGTGG	128
		Reverse	TTGCAGTTAGGTGACGCTGA	128
TLR4	NM_001310413.1	Forward	GACCTCCAGCACACGAAGTTAGAC	165
		Reverse	GGAGTTGCCTGCCATCTTGAGC	165
NLRP3	MH373356.1	Forward	CGCTGAACGAGGACGCACTG	124
		Reverse	TGGAAGGGTAGTCGGGACATAGC	124
TXNIP	XM_032204531.1	Forward	GCTGCCAAGAAGGAGAAGAAGGTG	130
		Reverse	TGTTCTCGAAGTCGGCGTTGATG	130
Caspase-1	XM_027446016.1	Forward	GCGGAACCAAGAGCAGAGATGAG	130
		Reverse	CCACGGCAGGACTGGATAATAACC	130
NF-κB	NM_001310777.1	Forward	GGAGCAGTGGCGGTGTCAAC	126
		Reverse	AGTGCAGTTCATGTCATCGGTCTC	126
p53	MT210098.1	Forward	CCATACGCAAGACGCCAAGGAG	126
		Reverse	AATCTTCACCATGTCCGTGCTGTC	126
β-actin	EF667345.1	Forward	ATGTCGCCCTGGATTTCG	62
		Reverse	CACAGGACTCCATACCCAAGAA	62

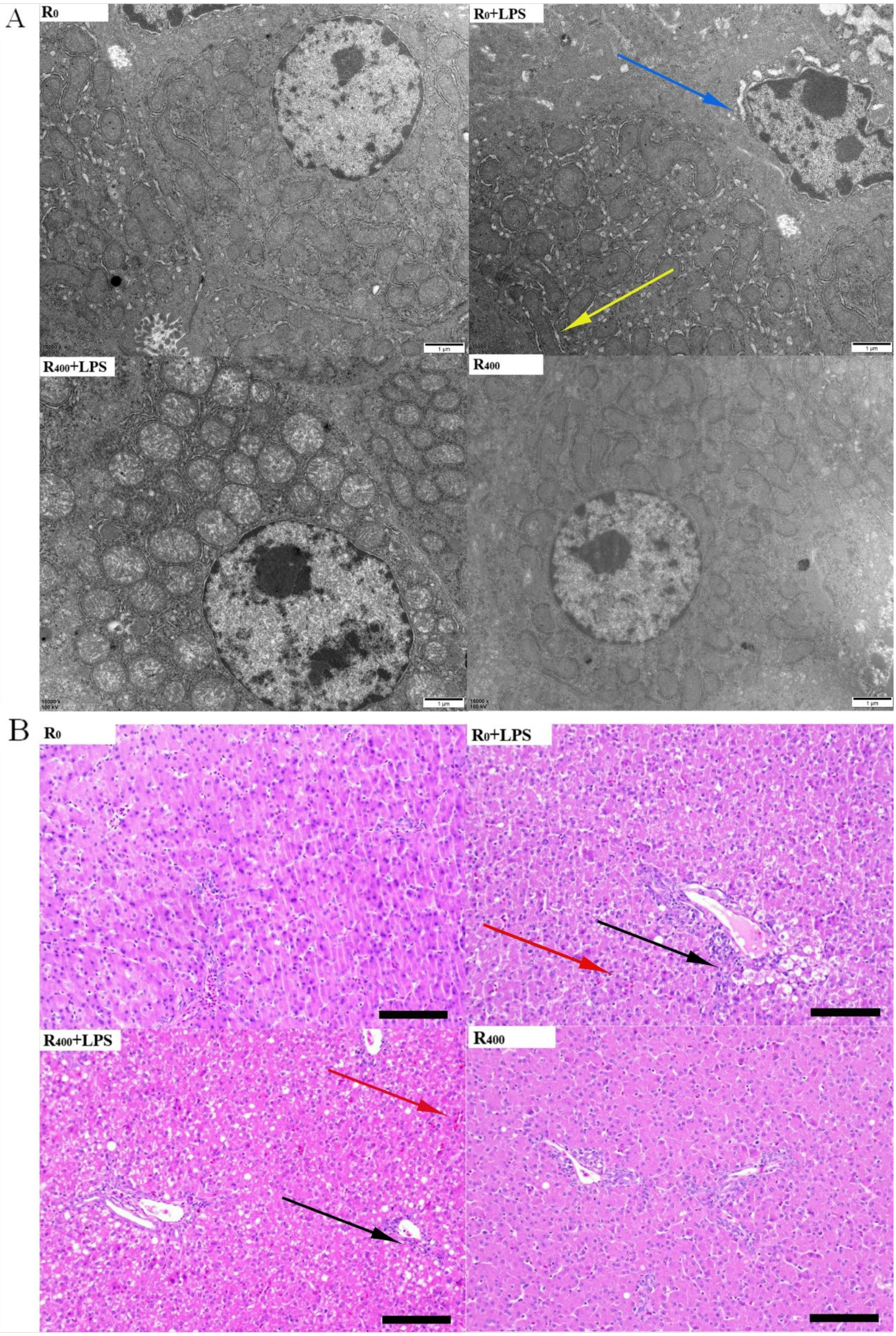

Figure S1. Effect of RES on the liver morphology and inflammatory response in duck ileum challenged by LPS. (**A**) Scanning electron microscope of duck liver tissue. The blue arrowhead indicated the shrinkage of nucleus, and the yellow arrowhead indicated the mild endoplasmic reticulum dilatation. (**B**) Representative photomicrographs of hematoxylin-eosin stained issue in liver (magnification, 200×; scale bars, 200 μm). The black arrowhead indicated the gathered inflammatory cell, and the red arrowhead indicated the grossly visible hemorrhage. R_0: the group fed with the corn-soybean basal diet; R_{400}: the group fed with the corn-soybean basal diet supplemented with 400 mg/kg

RES; R_0 + LPS: the group fed with the corn-soybean basal diet and injected with 5 mg LPS/kg duck body weight; R_{400} + LPS: the group fed with the corn-soybean basal diet supplemented with 400 mg/kg RES and injected with 5 mg LPS/kg duck body weight; (the same below).

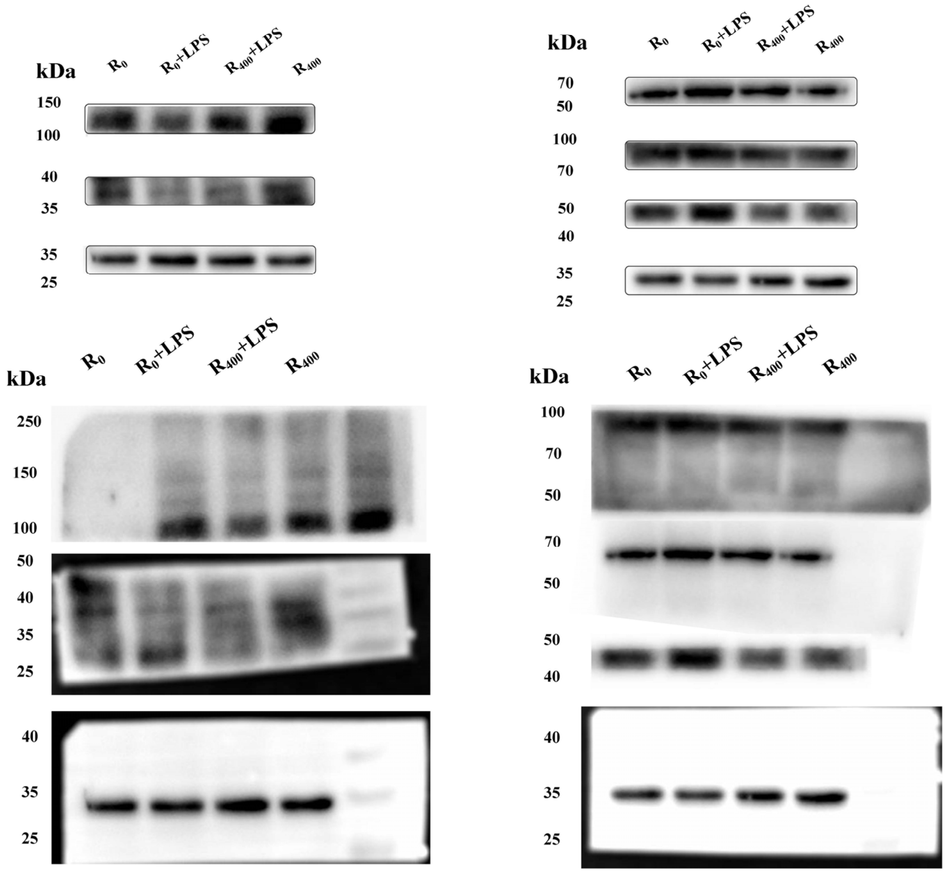

Figure S2. Original Western Blot figures.
